# Habitat Variability and Ethnic Diversity in Northern Tibetan Plateau

**DOI:** 10.1038/s41598-017-01008-8

**Published:** 2017-04-20

**Authors:** Xin Jia, Harry F. Lee, Mengchun Cui, Chao Liu, Lin Zeng, Ricci P. H. Yue, Yang Zhao, Huayu Lu

**Affiliations:** 1 School of Geographic and Oceanographic Sciences, Nanjing University, Nanjing, Jiangsu Province 210023 China; 2 Department of Geography, The University of Hong Kong, Hong Kong, SAR China; 3 School of Foreign Languages, Southeast University, Nanjing, Jiangsu Province 210096 China

## Abstract

There are 56 officially-recognized ethnic groups in China. However, the distinct geographic patterns of various ethnic groups in relation to the physical environment in China have rarely been investigated. Based on the geo-referenced physical environmental parameters of 455 Han, Tu, Hui, Salar, Mongolian, and Tibetan communities in Qinghai, we found that the communities could be statistically demarcated by temperature and aridity threshold according to their ethnicity, implying that the geographic distribution of each ethnic group is mediated by the physical environment. We also observed that the habitat of each ethnic group is ecologically compatible with current subsistence strategies. Tibetans settle in cold and humid high-altitude regions owing to the cultivation of highland barley and the breeding of yak, dzo, Tibetan sheep and Tibetan goat. Mongolians survive by animal husbandry in cold and dry grassland areas. Han and Tu people settle in the Huangshui River Valley, which offers relatively humid climate and flat land for agriculture. Hui and Salar people occupy the Yellow River Valley with its relatively arid environment and grassland vegetation suitable for animal breeding. Our findings offer a new perspective in explaining the geographic patterns and the varieties of ethnic groups in China and elsewhere.

## Introduction

Cultural diversity is one of the key areas of interest in anthropological research. Scholars often ask why diversification occurs and how the subsequent diversity is maintained^1^. China is home to 56 officially-recognized ethnic groups, and scholarly studies primarily focus on the differences among various ethnic groups and on their diverse origins^2, 3^, cultural development^4–6^, or genetic make-ups^7–9^. However, the geographic distribution of various ethnic groups in relation to the physical environment has rarely been systematically investigated. Given that ethnic groups in China exhibit strong geographic patterns, it is of interest to determine whether the patterns are accidental or attributable to physical environmental factors. We seek to investigate this topic in this study, as the associated findings may offer a new perspective in explaining the distribution and the variety of ethnic groups in China and elsewhere.

The environment of the Tibetan Plateau, with its high altitude, low temperatures, and lower availability of oxygen, makes it an arduous place for human habitation. While the Tibetan Plateau shares 20.03% of China’s territory, only 0.65% of the country’s population is settled there^10^. The first arrival of humans at the Tibetan Plateau can be traced back to 21,000 years ago^11^, and humans only settled permanently on the northern Tibetan Plateau after 8,000 cal. yr BP^12, 13^. A large number of humans migrated to the higher parts of the Tibetan Plateau along the Yellow River^14, 15^, a move that was probably facilitated by barley planting and sheep breeding^15–17^. Tea was introduced to the Tibetan Plateau through the Silk Road around 1,800 cal. yr BP^18^. Along with the communication and integration between the Tibetans and others around the Tibetan Plateau, diverse ethnic groups gradually formed during the Yuan-Ming-Qing Dynasties (AD1271–1911)^19^, including the Han, Tibetan, Mongolian, Hui, Salar, and Tu.

**Figure 1:**
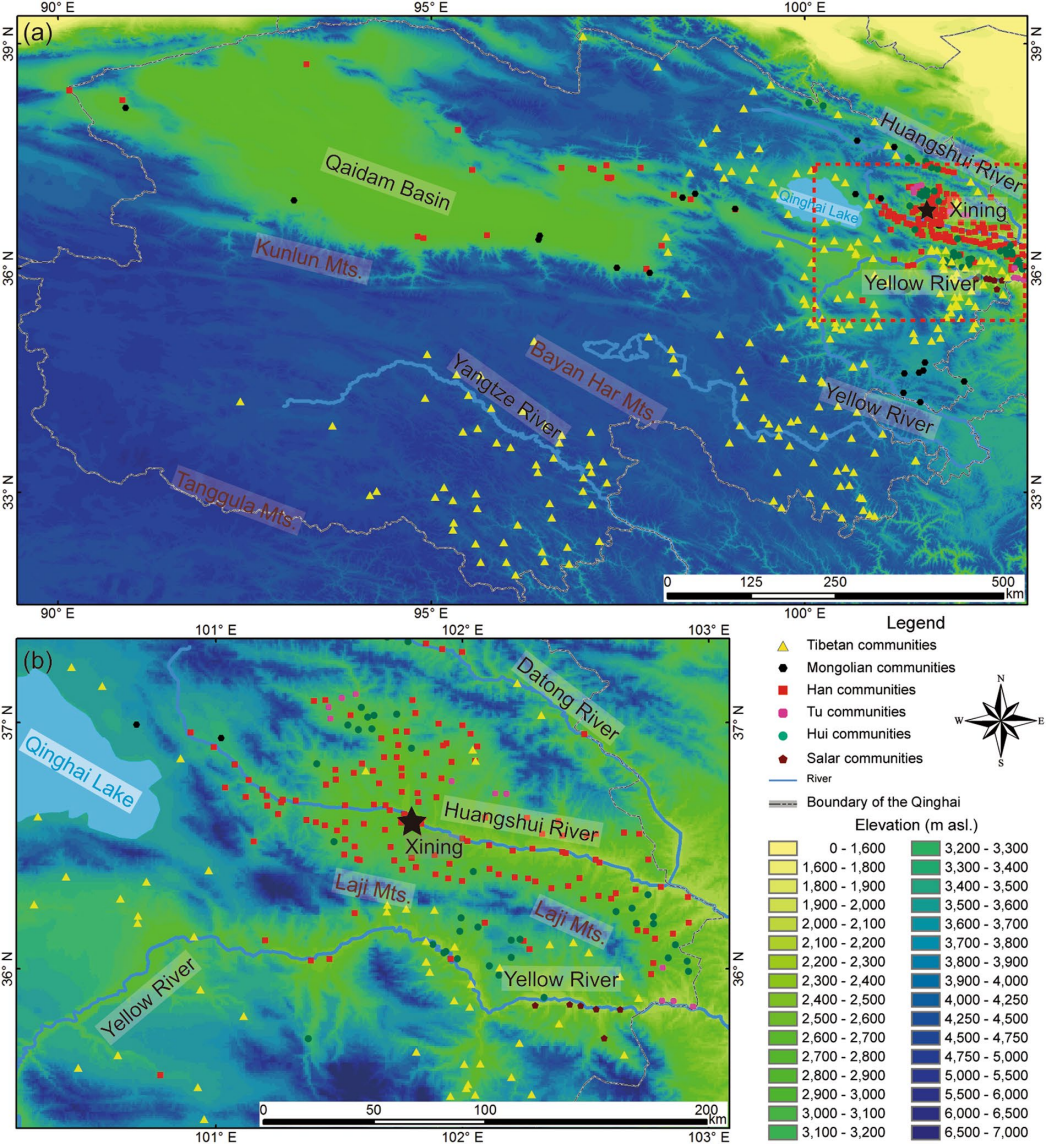
Distribution of the communities of major ethnic groups in Qinghai. (**a**) Map of Qinghai; (**b**) Map of Hehuang Region. The maps were generated in ArcGIS version 10.1 (www.esri.com/software/arcgis). The topographic information was obtained from the Digital Elevation Model (DEM) with a resolution of 90 m × 90 m, which was downloaded from the Consortium for Spatial Information (CGIAR-CSI) (http://srtm.csi.cgiar.org/).

Qinghai (89°35′–103°04′E, 31°9′–39°19′N, 1,650–6,860 m a.s.l.) is located in northwestern China and the northern part of the Tibetan Plateau. It covers an approximate area of 722,300 km^2^ (Fig. 1a). The region also overlaps with the northwestern fringe of the Asian Summer Monsoon, and hence, features a transitional zone between semi-humid and arid climates, with mean annual precipitation and evaporation ranged between 15.5–732.6 mm and 1,096.4–3,008.0 mm, respectively. Owing to the high altitude of the Tibetan Plateau, glacier meltwater nourishes the upper reaches of the Yangtze River, Yellow River, and Mekong River (called Lancang River in China). Numerous lakes, such as Qinghai Lake, Nam Co, and Siling Co, scatter around the southern and eastern Tibetan Plateau. They receive water from the abundant rainfall which is collected in some depressions. Aridity gradually increases from the northwest to the southeast in Qinghai. Qaidam Basin is the driest place in Qinghai. The huge elevation differences in the Tibetan Plateau (1,950–8,844 m a.s.l.) result in deep temperature gradients. The mean annual temperature is ranged between −5.1–8.3 °C, and the coldest spots appear in mountainous area. Subject to local climatic conditions, meadow, marsh, and grassland dominate in eastern Qinghai, while widely dispersed deserts occur in the Qaidam Basin in the northwest. In addition, farmland vegetation is distributed along the banks of the Hehuang Region in the northeast. Subject to the unique geographic setting of Qinghai, the huge habitat diversity there is unique among all the regions in China.

As per China’s Sixth Census in 2010, there are 55 ethnic groups distributed unevenly in Qinghai. In the present study, we based on 455 communities of the six most heavily represented ethnic groups in Qinghai, including the Han, Tibetan, Hui, Tu, Salar, and Mongolian groups, to investigate the possible connection between ethnic groups’ distribution and physical environmental factors. These are also the only ethnic groups in Qinghai that have population over 10,000. Our classification of ethnic groups was based on their dominant population stated in chronographs.

## Results

**Figure 2:**
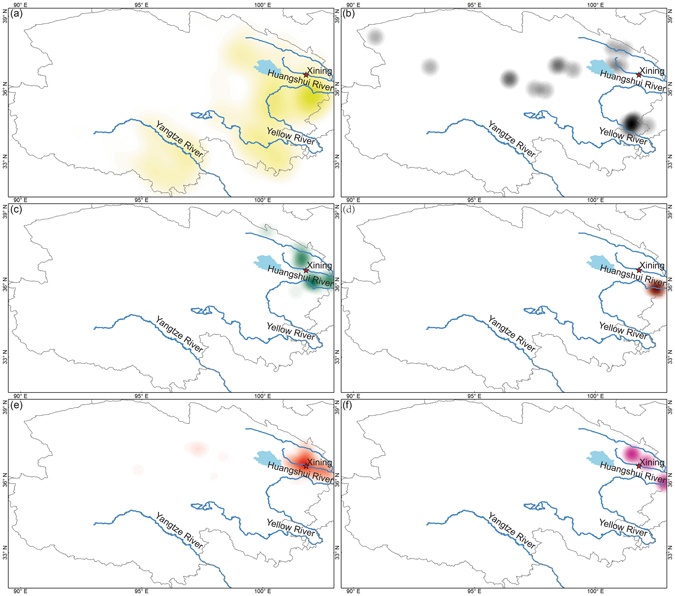
Density distribution of the communities of major ethnic groups in Qinghai. (**a**) Tibetan; (**b**) Mongolian; (**c**) Hui; (**d**) Salar; (**e**) Han; (**f**) Tu. The maps were generated in ArcGIS version 10.1 (www.esri.com/software/arcgis).

The density maps of the communities of each of the major ethnic groups reveal some notable patterns about their geographic concentration (Fig. 2). Tibetan communities are mostly concentrated in Huangnan, as well as Hainan and Golog Tibetan Autonomous Prefectures in the Yellow River Basin, Yushu Tibetan Autonomous Prefecture in the Yangtze headwater region, and the eastern margin of Haixi Mongolian-Tibetan Autonomous Prefecture in eastern Qaidam Basin (Fig. 2a). The distribution of Mongolian communities is dispersed, and small clusters are found in Henan Mongol Autonomous County, southern Huangnan Tibetan Autonomous Prefecture, and the headwater of the Yellow River (Fig. 2b). Clusters of Hui communities are found in the Yellow River (Hualong Hui and Minhe Hui-Tu Autonomous Counties) and the Huangshui River (Datong Hui-Tu Autonomous County) Basins (Fig. 2c). Salar communities are distributed only in Xunhua Salar Autonomous County in the Yellow River Basin (Fig. 2d). Han communities concentrate around Xining City in the Huangshui River Basin (Fig. 2e). Tu communities locate in the Guanting Basin (within the Yellow River Basin), Datong Hui-Tu and Huzhu Tu Autonomous Counties (within the Huangshui River Basin) (Fig. 2f).

**Figure 3:**
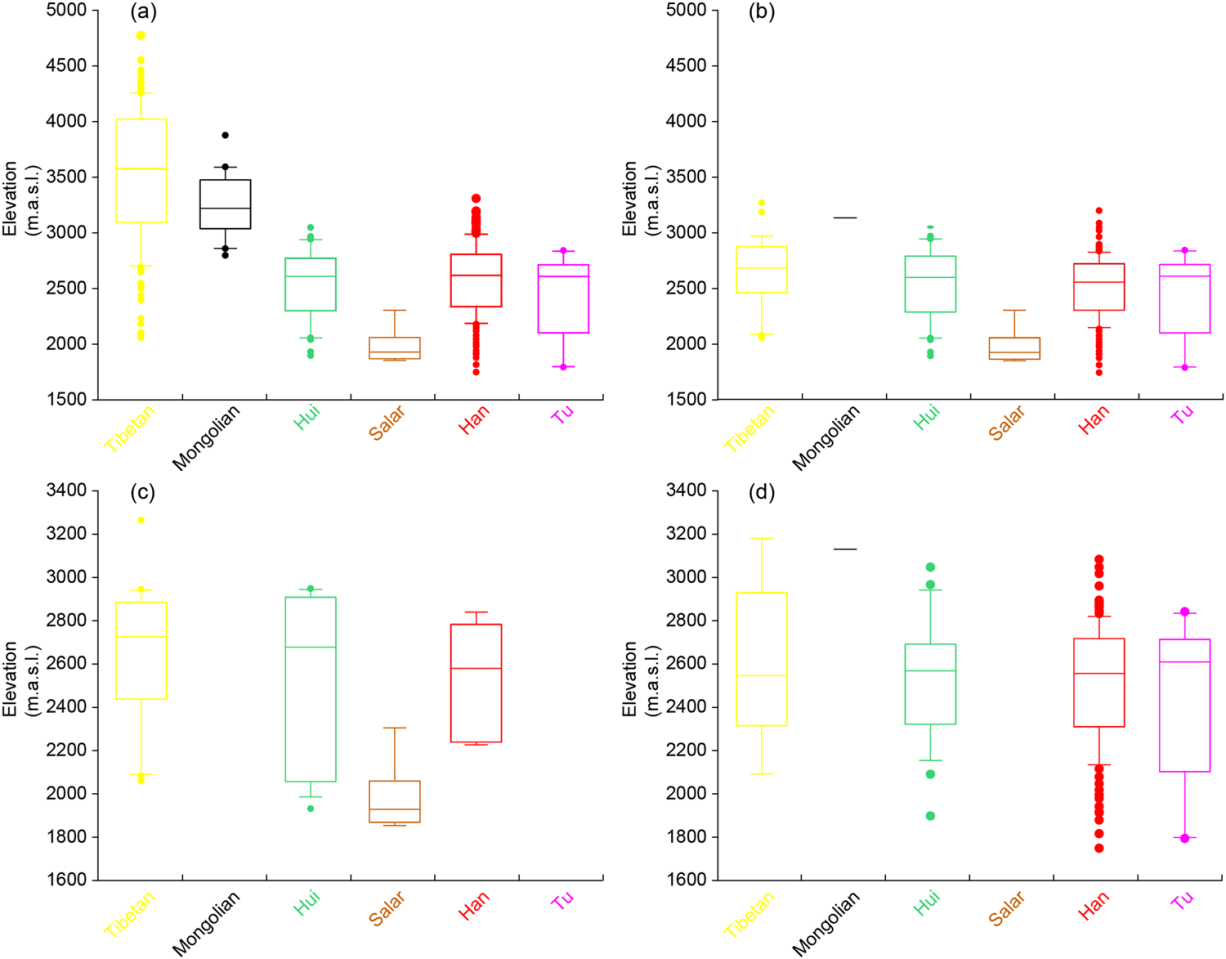
Box diagrams of the attitude of the communities of major ethnic groups in various regions in Qinghai. (**a**) The whole of Qinghai; (**b**) Hehuang Region; (**c**) Yellow River Valley; (**d**) Huangshui River Valley. The diagrams were drawn in Adobe Illustrator version CS6 (http://www.adobe.com/).

The altitude of each community is represented by the elevation of its administrative center (Fig. 3). We found that 53.2% of the communities are set at 2,000–3,000 m a.s.l., while 30.8% of the communities found are at 3,000–4,000 m a.s.l. Different ethnic groups locate in different altitudes. Han, Tu, Hui, and Salar communities dominate the region below 3,000 m a.s.l., while Mongolian and Tibetan communities dominate the region above 3,000 m a.s.l. The cold resistance of barley and sheep helped humans settle above 2,000 m a.s.l. around 3,600 cal. yr B.P.^15^. Given that elevation is closely associated with local climate and biomass, we proceeded to compare the communities’ locational environmental characteristics among various ethnic groups.

**Figure 4:**
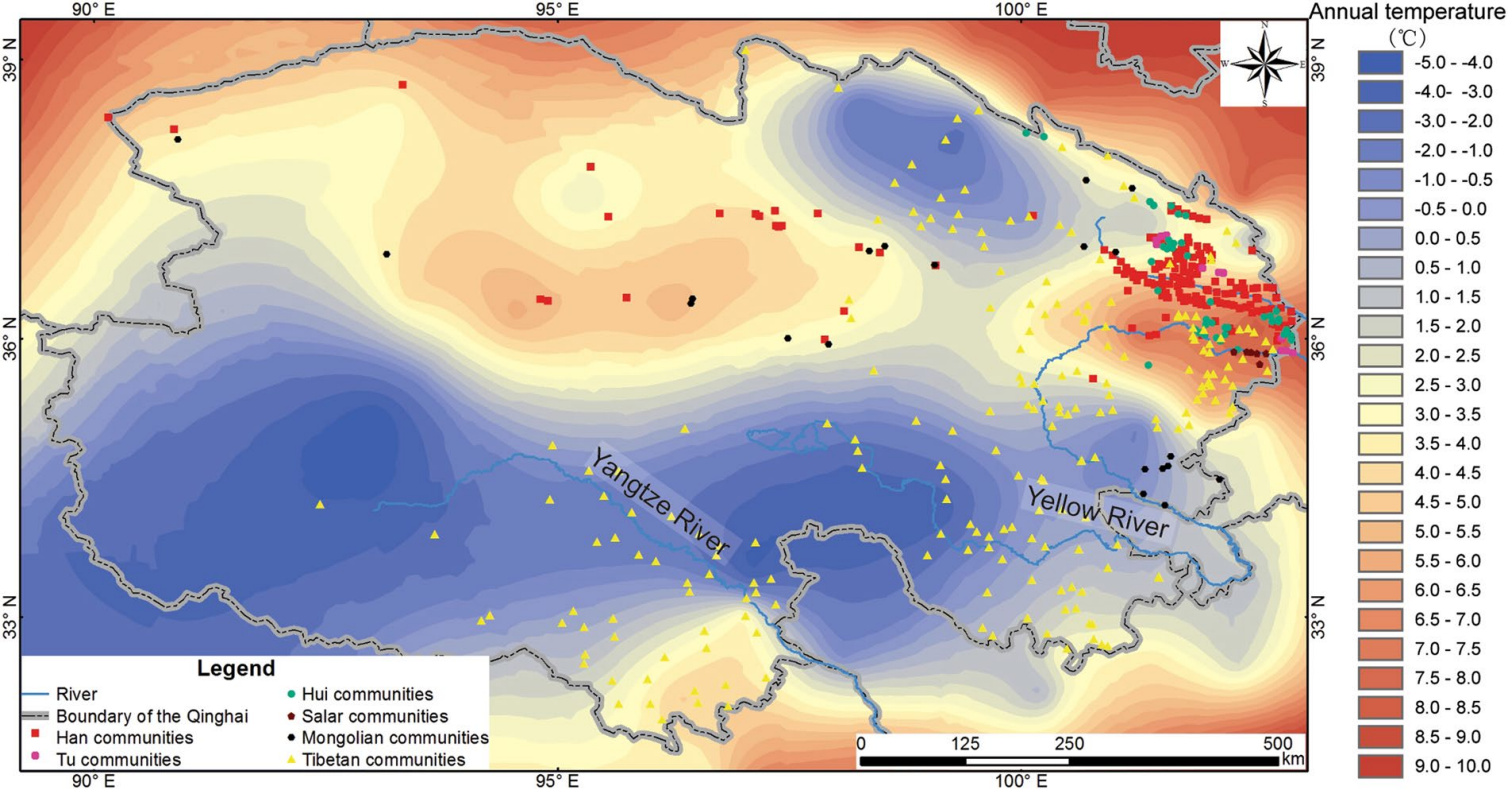
Mean annual temperature and the distribution of the communities of major ethnic groups in Qinghai. The temperature data were retrieved from China Meteorological Administration (http://data.cma.cn/). The map was generated in ArcGIS version 10.1 (www.esri.com/software/arcgis).

We found that the communities’ mean annual temperature and mean aridity threshold among various ethnic groups are significantly different (p < 0.05) (Table 1). The mean annual temperature associated with the Tibetan communities is the lowest among those of the major ethnic groups in Qinghai (1.95 ± 6.13 °C) (Fig. 4, Table 2), owing to their high altitude locations (3,517 ± 1,255 m a.s.l.) (Fig. 3). Statistically, their mean annual temperature is significantly lower than that of the Han, Tu, Hui, and Salar communities (p < 0.05) (Table 2).

**Table 1:** One-way ANOVA of the mean annual temperature and mean annual aridity threshold of the communities of major ethnic groups in Qinghai.

	Ethnic group						F	df^a^	df ^b^
	Han	Tu	Hui	Salar	Mongolian	Tibetan			
**Qinghai**									
Mean annual temperature	4.93	4.98	5.66	7.77	2.25	1.95	43.58*	5	449
Mean annual aridity threshold	7.20	3.51	4.24	4.10	16.46	4.04	5.42*	5	449
No. of communities	163	11	42	6	20	213			
**Qinghai without Qaidam Basin**									
Mean annual temperature	5.02	4.98	5.66	7.77	1.52	1.97	42.79*	5	420
Mean annual aridity threshold	4.04	3.51	4.24	4.10	4.17	3.98	0.29	5	420
No. of communities	146	11	42	6	14	207			

^a^Degree of freedom (between groups).

^b^Degree of freedom (within groups).

^*^Via One-way ANOVA analysis, significant at 0.05 level (P < 0.05).

**Table 2:** Tukey HSD test of the mean annual temperature and mean annual aridity threshold of the communities of major ethnic groups in Qinghai.

Ethnic group	Compared ethnic group	Qinghai		Qinghai without Qaidam Basin	
		Temp mean diff	Arid mean diff	Temp mean diff	Arid mean diff
Han	Tu	−0.06	3.69	0.04	0.53
	Hui	−0.74	2.95	−0.64	−0.21
	Salar	−2.84*	3.10	−2.75	−0.07
	Mongolian	2.68*	−9.27*	3.50*	−0.14
	Tibetan	2.97*	3.15	3.06*	0.06
Tu	Han	0.06	−3.69	−0.04	−0.53
	Hui	−0.68	−0.74	−0.68	−0.74
	Salar	−2.78	−0.60	−2.78	−0.60
	Mongolian	2.74*	−12.96*	3.47*	−0.67
	Tibetan	3.03*	−0.54	3.02*	−0.47
Hui	Han	0.74	−2.95	0.64	0.21
	Tu	0.68	0.74	0.68	0.74
	Salar	−2.10	0.14	−2.10	0.14
	Mongolian	3.42*	−12.22*	4.15*	0.07
	Tibetan	3.71*	0.20	3.70*	0.26
Salar	Han	2.84*	−3.10	2.75	0.07
	Tu	2.78	0.60	2.78	0.60
	Hui	2.10	−0.14	2.10	−0.14
	Mongolian	5.52*	−12.36	6.25*	−0.07
	Tibetan	5.82*	0.06	5.80*	0.12
Mongolian	Han	−2.68*	9.27*	−3.50*	0.14
	Tu	−2.74*	12.96*	−3.47*	0.67
	Hui	−3.42*	12.22*	−4.15*	−0.07
	Salar	−5.52*	12.36	−6.25*	0.07
	Tibetan	0.30	12.42*	−0.45	0.19
Tibetan	Han	−2.97*	−3.15	−3.06*	−0.06
	Tu	−3.03*	0.54	−3.02*	0.47
	Hui	−3.71*	−0.20	−3.70*	−0.26
	Salar	−5.82*	−0.06	−5.80*	−0.12
	Mongolian	−0.30	−12.42*	0.45	−0.19

*Via Tukey HSD test, significant at 0.05 level (P < 0.05).

**Figure 5:**
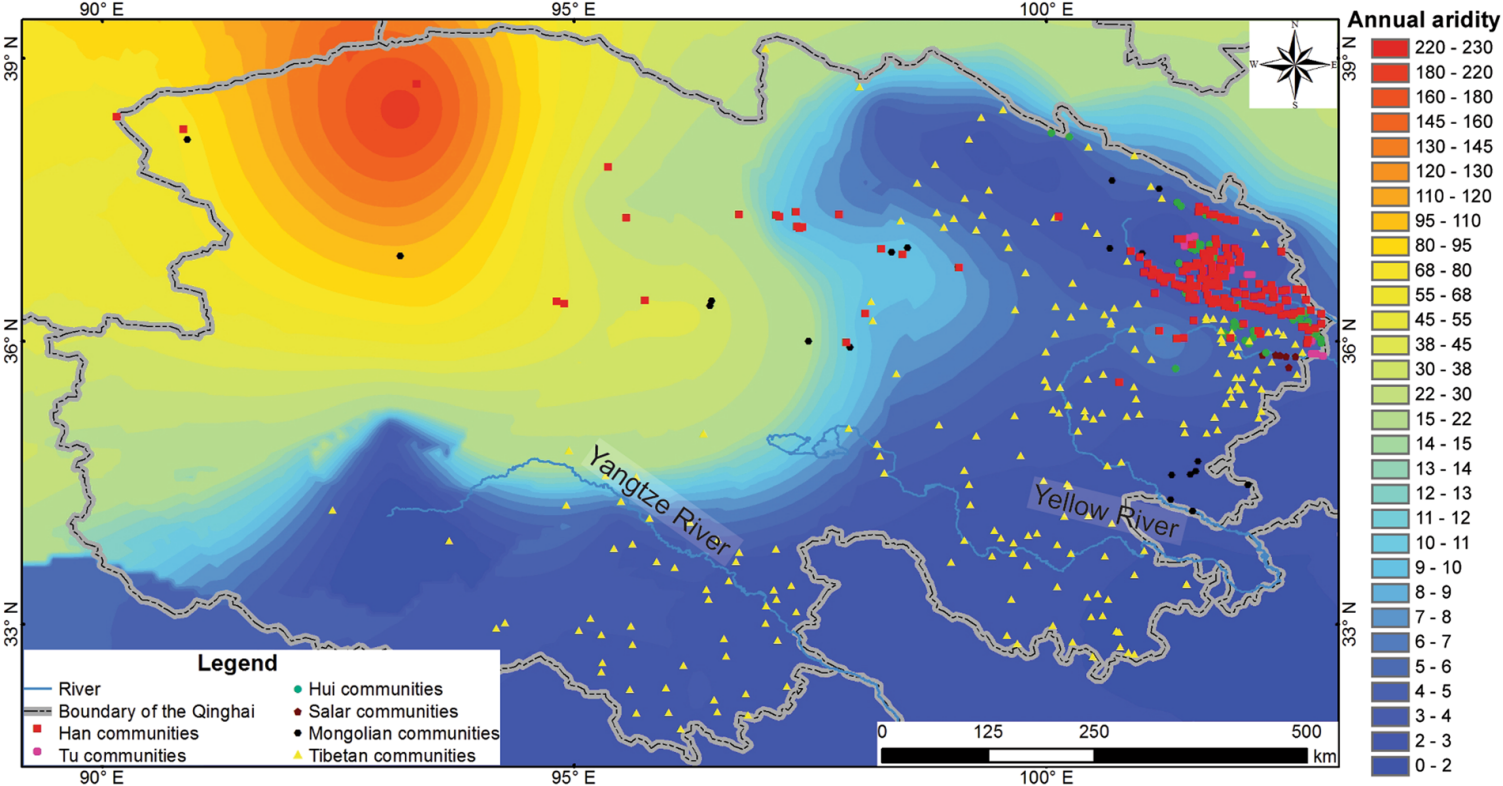
Mean annual aridity threshold and the distribution of the communities of major ethnic groups in Qinghai. The aridity threshold data were derived from the evaporation and precipitation datasets (evaporation divided by precipitation) that were retrieved from China Meteorological Administration (http://data.cma.cn/). The map was generated in ArcGIS version 10.1 (www.esri.com/software/arcgis).

The Mongolian communities are associated with the second lowest mean annual temperature (2.25 ± 2.78 °C) (Fig. 4, Table 2). Although the temperature is a bit higher than that of the Tibetan communities (1.95 ± 6.13 °C, it is significantly lower than that of the other four ethnic groups’ communities (5.14 ± 3.06 °C) (p < 0.05) (Fig. 4, Table 2). On the other hand, the mean aridity threshold of Mongolian communities (16.46 ± 88.77) is significantly higher than that of other ethnic groups’ communities (6.37 ± 166.47) (p < 0.05) (Fig. 5, Table 2). This may be caused by the extreme aridity threshold values associated with those communities in the Qaidam Basin. We excluded all the communities in the Qaidam Basin and then recreated our analysis. No significant difference of the communities’ aridity threshold values among all ethnic groups was observed (p > 0.05) (Table 2).

We further focused on the Hehuang Region (Fig. 1b), because 88.7% of the communities in Qinghai are located there. Based on the type of the communities, six ethnic groups were combined to four groups for further statistical comparison, namely agricultural (Han and Tu), agri-nomad (Hui and Salar), pastoral (Mongolian), and plateau (Tibetan). We found that the communities’ mean annual temperature and mean aridity threshold among the above groups are significantly different (p < 0.05) (Table 3). The mean annual temperature (6.06 ± 2.14 °C) and the mean aridity threshold (4.15 ± 1.14) of the agri-nomad communities are higher than the agricultural ones (5.11 ± 3.08 °C and 3.86 ± 2.64) (p < 0.05) (Table 4). Agri-nomad communities mainly scatter around the southern slope of the Laji Mountain in the Yellow River Valley (Fig. 2c and d). The more intense solar radiation and stronger evaporation there result in relatively higher temperature and aridity threshold values in the Yellow River Valley than those in the Huangshui River Valley, where the agricultural communities locate (Fig. 5, Table 4).

**Table 3:** One-way ANOVA of the mean annual temperature and mean annual aridity threshold of the communities of major ethnic groups (combined) in Hehuang Region.

Ethnic group (combined)	Agricultural	Agri-nomad	Pastoral	Plateau	F	df^a^	df ^b^
Mean annual temperature	5.12	6.06	2.39	6.03	8.07*	3	226
Mean annual aridity threshold	3.86	4.15	3.58	4.2	4.29*	3	226
No. of communities	151	46	3	30			

^a^Degree of freedom (between groups).

^b^Degree of freedom (within groups).

*Via One-way ANOVA analysis, significant at 0.05 level (P < 0.05).

## Discussion

### Linkage between environmental factors and the geographic distribution of ethnic groups

Owing to the unique geographic setting of the Tibetan Plateau, located at the transition zone between semi-humid and semi-arid climate, Qinghai is traversed by the ecotone between agriculture and animal husbandry^20^. Association between human settlement patterns and subsistence strategies in our study region in pre-historic^15–17^ and historic periods^21^ has been evidenced by archaeological and historical records. In the present study, we found that the geographic pattern of the communities of various ethnic groups in Qinghai still exhibits a close connection with physical environmental factors. Also, the connection matches very closely with the current subsistence strategies of various ethnic groups.

The Tibetan is a plateau race. Tibetans live in the cold high-altitude region in Qinghai, and provide for themselves with highland barley, yak, dzo, Tibetan sheep, and Tibetan goat. Also, in Tibet, Gansu, Sichuan, and Yunnan, Tibetans mainly locate in cold high-altitude regions. Highland barley, the primary subsistence crop of the Tibetans, is suitable in the high-altitude zone owing to its genetic tolerance to low temperature (with a semi-lethal low temperature of −4.8–18.4 °C)^22^. Yak^23^, dzo, Tibetan sheep^24^, and Tibetan goat^25^ are cold-resistant livestock complimentary to highland barley, supplying meat, eggs, and dairy products to the Tibetans. Tibetans carry a unique gene (HIF2A/EPAS1 and EGLN1) adapted to low temperature^26, 27^ perhaps as a result of the limited food resources. Such genetic make-up probably enables the Tibetans to survive in the cool high-altitude regions. Still, a biological threshold can be observed. There are very few people living in the coldest part of Qinghai, and −5 °C is probably the minimum mean annual temperature value allowing for large-scale human communities there (Fig. 4).

**Figure 6:**
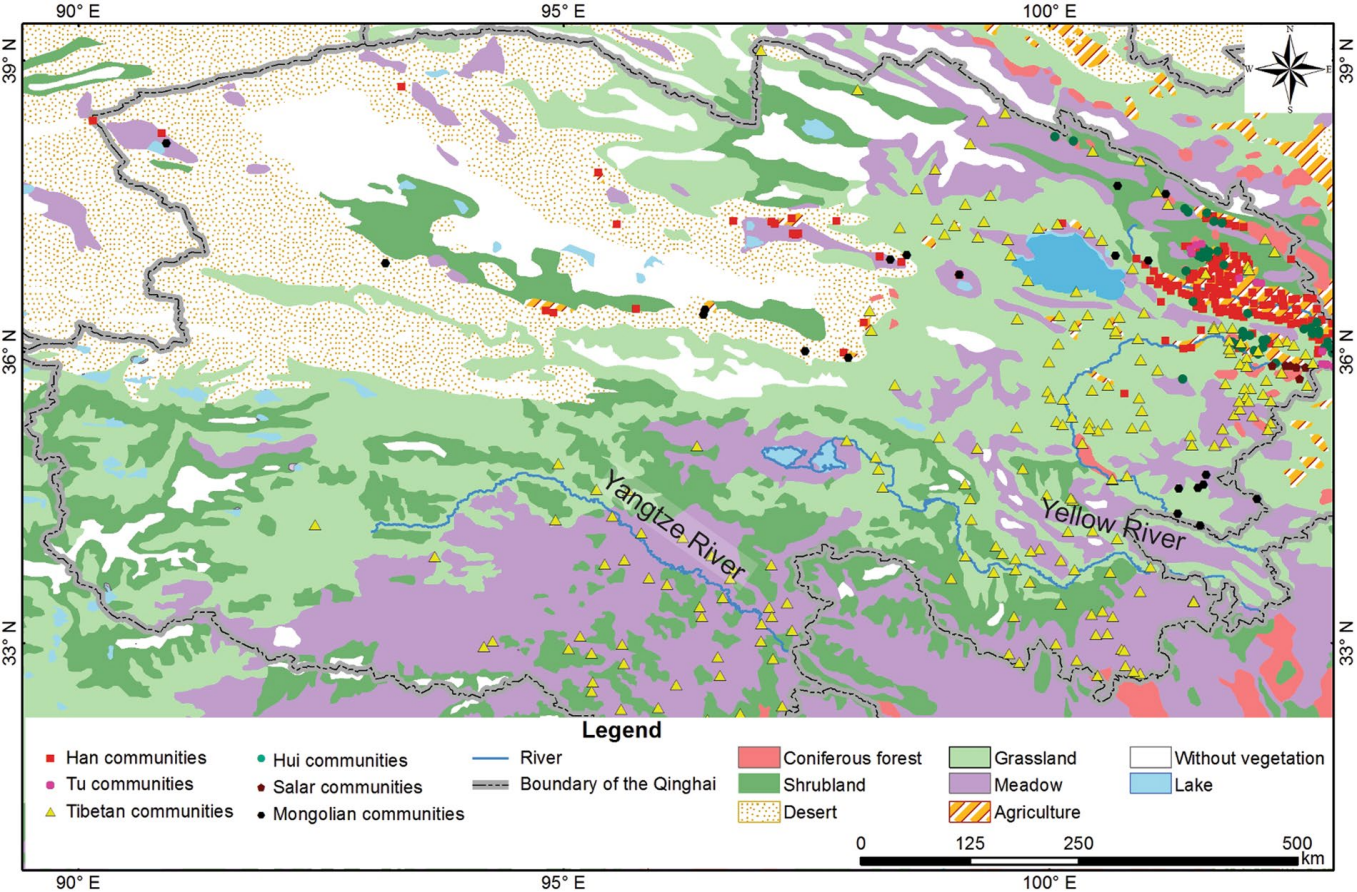
Vegetation cover and the distribution of the communities of major ethnic groups in Qinghai. The vegetation cover data set was provided by Cold and Arid Regions Science Data Centre at Lanzhou (http://westdc.westgis.ac.cn). The map was generated in ArcGIS version 10.1 (www.esri.com/software/arcgis).

The Mongolians subsist with animal husbandry on grassland or meadow, and are widely distributed in central and eastern Inner Mongolia. Such vegetation, which is grown in lower temperatures and relatively arid environments, fits into the animal husbandry activities of the Mongolians. There are eight Mongolian communities distributed in the grassland, six in the meadow, five in the desert, and one in the shrub-land (Fig. 6). The grassland vegetation in the Henan Mongolia Autonomous County is similar to the one in the middle and the eastern parts of Inner Mongolia (Fig. 6). Vast grasslands support a large number of sheep and cattle, sustaining the husbandry of the Mongolian communities. In addition, some Mongolian communities spread along areas of alpine meadow vegetation, where the cattle and sheep can be fed on abundant pasture.

The diversity of ethnic groups in the Hehuang Region may be attributable to the substantial regional variation of the aridity threshold. Within the region, the relatively humid environment in the Huangshui River Valley ensures crop growth and hence, a large amount of cultivated land has been opened up in the valley (Fig. 6). The arid environment in the Yellow River Valley does not meet the water demand of crops and is only suitable for dry grassland, as well as animal husbandry activities (Fig. 6). The above disparity paves the way for the differentiation of subsistence strategies within the Hehuang Region. Agricultural communities plant wheat, potato, rape and other crops and settle in relatively humid flatland in the Huangshui River Valley. As agri-nomadic communities engage in both agriculture and animal husbandry, they occupy relatively arid grassland in the Yellow River Valley, where the husbandry that supplies red meat to the Hui and Salar is also supported.

In addition, the Huangshui River is a tributary of the Yellow River. Its erosion datum surface (1,580 m a.s.l.) locates where the Huangshui River feeds into the Yellow River. The Huangshui River bends in the valley owing to the small fall (4,200–1,580 m) and tardier down-erosion. Because of steady lateral erosion, the Huangshui River Valley is more flat (Fig. 1b) and more appropriate for crop cultivation in comparison to the Yellow River Valley (Fig. 6). The physical environment in the Huangshui River Valley is ideal for the Han and Tu in terms of subsistence strategy.

### Landscape diversity and the variety of ethnic groups

There are 5.6 million people in Qinghai. When compared with other provinces in China, it is not very populated in terms of population size and population density^10^. But, 55 ethnic groups are found there. There are six major ethnic groups (i.e., ethnic group with a population >1% of the provincial population) in Qinghai [Han (53.02% of the provincial population), Tibetan (24.44%), Hui (14.83%), Tu (3.63%), Salar (1.90%), and Mongolian (1.77%)]^10^, which are more than those in nearby provinces, including: Xinjiang (Han, Uyghur, Kazak, and Hui), Tibet (Tibetan and Han), Mongolia (Han, Mongolian, and Manchu), Gansu (Han, Hui, Dongxiang, and Tibetan), Sichuan (Han, Yi, and Tibetan), and Ningxia (Han, Hui, and Manchu). Besides, in terms of the proportion in provincial population, the composition of ethnic groups in Qinghai is more balanced when compared with that in nearby provinces. For instance, in Tibet, 90.48% of the total population is composed of Tibetans other than Han (8.17%). In Inner Mongolia, Gansu, and Sichuan, Han people share about 79.54%, 90.58%, and 93.90% of the provincial population, respectively. However, in Xinjiang, which consists of the Tian Shan mountain range and the Junggar and Tarim Basins and is marked by huge elevation differences, the composition of ethnic groups is rather balanced (i.e., Han 40.48%; Uyghur 45.84%; Kazak 6.50%; Hui 4.51%), which is comparable to the ethnic make-up of Qinghai^10^ (Table S1). Furthermore, the habitat of the six major ethnic groups in Qinghai corresponds to their subsistence strategies. In terms of the number of communities, agricultural ethnic groups share about 38.24%, agri-nomadic ethnic groups share about 10.55%, pastoral ethnic groups share about 4.40%, while plateau ethnic groups share about 46.81%. The Plateau (Tibetan) ethnic group is mainly distributed in the high attitude area (above 4,000 m a.s.l.). As Tibetans have accustomed themselves to the cold-humid rather than the cold-arid environment, they are not dominant in Xinjiang and Inner Mongolia (Table S1). On the other hand, although the cold-arid environment is suitable for animal husbandry, it is not favorable for steppe, leading to the scarcity of the pastoral (Mongolian) ethnic group in Gansu, Sichuan, and Tibet (Table S1). The agricultural (Han, Tu, Kazak, and Yi) ethnic group is distributed in all of the provinces in northwestern China. Yet, subject to the environmental constraints of agriculture, it is hard for agricultural ethnic group to live in areas with high elevation. Hence, only 8.17% of the population in Tibet belongs to agricultural ethnic group (Table S1). In addition, the agri-nomadic (Hui, Uyghur, Dongxiang and Salar) ethnic group is scarce in Sichuan, Mongolia, and Tibet (Table S1) for different reasons. Southward migration of the pastoral ethnic group to Sichuan is hampered by the humid environment, while the development of low-altitude agriculture in Mongolia and Tibet is restricted by the cold environment. In addition, a unique ethnic group called Manchu lives in the humid forest area, and they sustain their living by hunting and gathering. The four types of subsistence strategies in Qinghai probably arise from the variety of physical environments there. Different habitats are characterized by different elevation, temperature, and aridity threshold, while the presence or absence of certain geographical features in some regions probably makes certain subsistence strategies more or less feasible there.

**Table 4:** Tukey HSD test of the mean annual temperature and mean annual aridity threshold of the communities of major ethnic groups (combined) in Hehuang Region.

Ethnic group (combined)	Compared ethnic group (combined)	Temp mean diff	Arid mean diff
Agricultural	Agri-nomad	−0.95*	−0.29*
	Pastoral	2.72*	0.28
	Plateau	−0.92*	−0.34*
Agri-nomad	Agricultural	0.95*	0.29*
	Pastoral	3.67*	0.57
	Plateau	0.03	−0.05
Pastoral	Agricultural	−2.72*	−0.28
	Agri-nomad	−3.67*	−0.57
	Plateau	−3.64*	−0.62
Plateau	Agricultural	0.92*	0.34*
	Agri-nomad	−0.03	0.05
	Pastoral	3.64*	0.62

*Via Tukey HSD test, significant at 0.05 level (P < 0.05).

Previous studies have built on environmental determinants such as precipitation, vegetation, and pathogens to account for cultural diversity at the global and continental levels^1, 28^. Our empirical findings show that the geographic distribution of ethnic groups in terms of their communities is related to physical environmental factors such as temperature, aridity threshold, and vegetation cover at the provincial level, indicating the possibility for the relationship to be downscaled to lower geographic levels.

At the global level, the present cultural diversity is a rich variety of hunter-gathers, pastoralists, agriculturalists, and more complex societies, revealing the possibility that the process of cultural diversification may be independent of subsistence^1^. Yet, at lower geographic levels, the landscape diversity in Qinghai is associated with the diverse subsistence strategies of ethnic groups and consequently determines their geographic distribution and variety. This is substantiated by the analogy of human genetics. People of some ethnic groups such as Tibetans and Han Chinese are basically genetically homogenous^29–31^, implying that they may have common origins. But, when they live in different environments, they equip themselves with different subsistence strategies for food selection and environmental adaptation, and have subsequently evolved into different cultures.

## Methods

### Data

Although the territories and the population size of communities have been changing over time, their ethnic grouping has been rather stable in the long-term. In this study, the ethnic grouping of communities was made according to their most dominant population stated in chronographs. Coordinates of the communities were obtained from Google Earth. The altitude of each community is represented by the elevation of its administrative center.

To quantify the physical environmental parameters of Qinghai, the mean annual temperature, precipitation, and evaporation data from 39 meteorological stations in Qinghai in 1980–2010 (http://data.cma.cn/) and the 1:4,000,000 vegetation map (http://westdc.westgis.ac.cn) (Fig. S1) were employed. And the mean annual aridity threshold was calculated by dividing the precipitation from the evaporation. In addition, the coordinates for those meteorological stations were obtained from Google Earth. Based on the above data inputs, the mean annual temperature, precipitation, and evaporation associated with each community were obtained via the Kriging Interpolation function in ArcGIS. The mean annual aridity threshold index of each community was derived from the resultant values of evaporation and precipitation (i.e., evaporation divided by precipitation).

### Statistical analysis

Based on the coordinates of the communities of each of the major ethnic groups, the spatial analytical tool *Point Density* in ArcGIS version 10.1 was employed for density analysis and the making of density maps. Under this method, a cluster (i.e., geographic concentration of communities) is determined by the number of communities of a particular ethnic group which fall within a neighborhood around each cell. In this study, the radius parameter of neighborhood was set as 2 km in calculating the geographic concentration of communities.

One-way ANOVA was used to test for significant differences between the means over a single dependent variable for two or more groups divided by an explanatory variable (i.e., independent factor). If F is significant, it can be concluded that there are differences in group means and thereby, the explanatory variable is proven to have an effect on the dependent variable^32^. In the present study, One-way ANOVA was employed to test whether the communities of various ethnic groups could be demarcated by environmental parameters (i.e., temperature and aridity threshold). The ethnic grouping of communities was entered as the independent ANOVA factor, while the value of each type of environmental parameter was entered as a dependent ANOVA factor. In addition, Tukey HSD test was used in conjunction with One-way ANOVA to identify which pairs of groups show statistically significant mean differences.

## Electronic supplementary material

Supplementary Information for Habitat Variability and Ethnic Diversity in Northern Tibetan Plateau
