# Supplementary material for: Habitat Variability and Ethnic Diversity in Northern Tibetan Plateau
Source: Sci Rep. 2017 Apr 20;7:918. doi: 10.1038/s41598-017-01008-8 (PMC5430525; doi:10.1038/s41598-017-01008-8)
Supplement: Supplementary file 1 — Supplementary Information for Habitat Variability and Ethnic Diversity in Northern Tibetan Plateau [file 41598_2017_1008_MOESM1_ESM.doc]

Supplementary Information for

**Habitat Variability and Ethnic Diversity in Northern Tibetan Plateau**

Xin Jia^1*^, Harry F. Lee^2^, Mengchun Cui^1^, Chao Liu^3^, Lin Zeng^1^, Ricci P.H. Yue^2^, Yang Zhao^1^ & Huayu Lu^1^

1 School of Geographic and Oceanographic Sciences, Nanjing University, Nanjing, Jiangsu Province, 210023, China;

2 Department of Geography, The University of Hong Kong, Hong Kong, SAR, China;

3 School of Foreign Languages, Southeast University, Nanjing, Jiangsu Province, 210096, China.

*Corresponding author. E-mail: jiaxin@nju.edu.cn

This Supplementary Text file includes:

Table S1

Figure S1

**Table S1. The composition of ethnic groups in the neighboring provinces of Qinghai.**

| Ethnic group | | China | Qinghai | Xinjiang | Inner Mongolia | Gansu | Sichuan | Tibet |
| --- | --- | --- | --- | --- | --- | --- | --- | --- |
| Total population | | 1,332,810,869 | 5,626,723 | 21,815,815 | 24,706,291 | 25,575,263 | 80,417,528 | 3,002,165 |
|  | |  |  |  |  |  |  |  |
| Han | No. | 1,220,844,520 | 2,983,521 | 8,829,994 | 19,650,665 | 23,164,817 | 75,509,724 | 245,263 |
|  | % | 91.60 | 53.02 | 40.48 | 79.54 | 90.58 | 93.90 | 8.17 |
|  |  |  |  |  |  |  |  |  |
| Mongolian | No. | 5,981,840 | 99,815 |  | 4,226,090 |  |  |  |
|  | % | 0.45 | 1.77 |  | 17.11 |  |  |  |
|  |  |  |  |  |  |  |  |  |
| Hui | No. | 10,586,087 | 834,298 | 983,015 |  | 1,258,641 |  |  |
|  | % | 0.79 | 14.83 | 4.51 |  | 4.92 |  |  |
|  |  |  |  |  |  |  |  |  |
| Tibetan | No. | 6,282,187 | 1,375,059 |  |  | 488,359 | 1,496,524 | 2,716,388 |
|  | % | 0.47 | 24.44 |  |  | 1.91 | 1.86 | 90.48 |
|  |  |  |  |  |  |  |  |  |
| Uyghur | No. | 10,069,346 |  | 10,001,302 |  |  |  |  |
|  | % | 0.76 |  | 45.84 |  |  |  |  |
|  |  |  |  |  |  |  |  |  |
| Manchu | No. | 10,387,958 |  |  | 452,765 |  |  |  |
|  | % | 0.78 |  |  | 1.83 |  |  |  |
|  |  |  |  |  |  |  |  |  |
| Yi | No. | 8,714,393 |  |  |  |  | 2,643,953 |  |
|  | % | 0.65 |  |  |  |  | 3.29 |  |
|  |  |  |  |  |  |  |  |  |
| Kazak | No. | 1,462,588 |  | 1,418,278 |  |  |  |  |
|  | % | 0.11 |  | 6.50 |  |  |  |  |
|  |  |  |  |  |  |  |  |  |
| Dongxiang | No. | 621,500 |  |  |  | 546,255 |  |  |
|  | % | 0.05 |  |  |  | 2.14 |  |  |
|  |  |  |  |  |  |  |  |  |
| Tu | No. | 289,565 | 204,412 |  |  |  |  |  |
|  | % | 0.02 | 3.63 |  |  |  |  |  |
|  |  |  |  |  |  |  |  |  |
| Salar | No. | 130,607 | 107,089 |  |  |  |  |  |
|  | % | 0.01 | 1.90 |  |  |  |  |  |

Source: The Population Census Office under the State Council, Department of population and employment statistics, National Bureau of Statistics. Tabulation on the 2010 population census of the People’s Republic of China. Beijing: Chinese Statistics Press (2012).


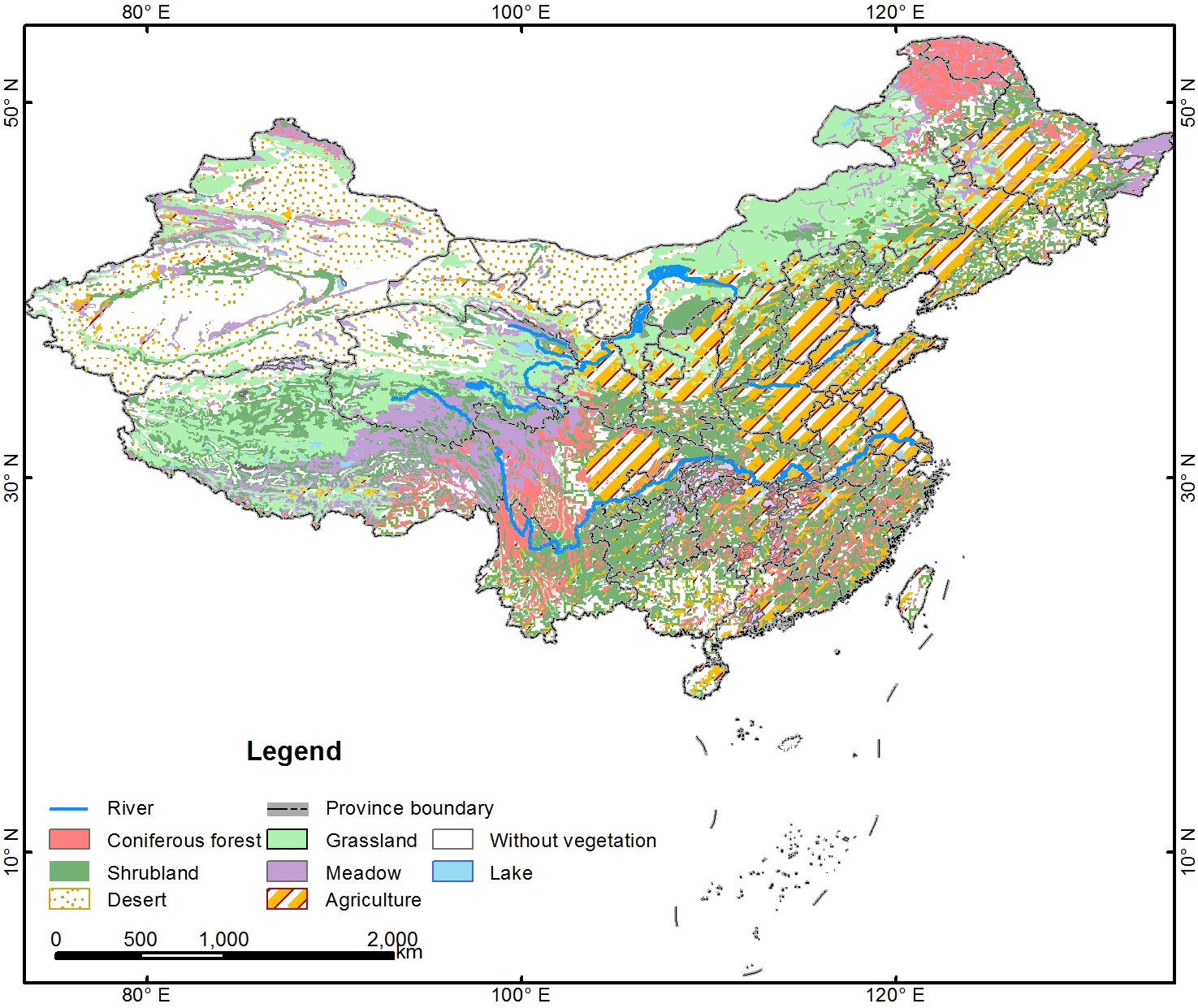


Source: The vegetation cover data set is provided by Cold and Arid Regions Science Data Center at Lanzhou ([http://westdc.westgis.ac.cn](http://westdc.westgis.ac.cn/)). The map is generated in ArcGIS version 10.1 ([www.esri.com/software/arcgis](http://www.esri.com/software/arcgis)). Scientific Reports remains neutral with regard to jurisdictional claims in published maps.

**Figure S1. Vegetation map of China.**
